# Adsorption of Carbon Dioxide and Nitrogen in Co_3_(ndc)_3_(dabco) Metal–Organic Framework

**DOI:** 10.3390/ijms25189951

**Published:** 2024-09-15

**Authors:** Rui Pedro Pinto Lopes Ribeiro, José Paulo Barbosa Mota

**Affiliations:** LAQV-REQUIMTE, Department of Chemistry, NOVA School of Science and Technology, NOVA University Lisbon, 2829-516 Caparica, Portugal; pmota@fct.unl.pt

**Keywords:** adsorbent, carbon capture, MOF, thermodynamics

## Abstract

Metal–organic frameworks (MOFs) are promising materials for processes such as carbon dioxide (CO_2_) capture or its storage. In this work, the adsorption of CO_2_ and nitrogen (N_2_) in Co_3_(ndc)_3_(dabco) MOF (ndc: 2,6-naphthalenedicarboxylate; dabco: 1,4-diazabicyclo[2.2.2]octane) is reported for the first time over the temperature range of 273–323 K and up to 35 bar. The adsorption isotherms are successfully described using the Langmuir isotherm model. The heats of adsorption for CO_2_ and N_2_, determined through the Clausius–Clapeyron equation, are 20–27 kJ/mol and 10–11 kJ/mol, respectively. The impact of using pressure and/or temperature swings on the CO_2_ working capacity is evaluated. If a flue gas with 15% CO_2_ is fed at 6 bar and 303 K and regenerated at 1 bar and 373 K, 1.58 moles of CO_2_ can be captured per kg of MOF. The analysis of the multicomponent adsorption of typical flue gas streams (15% CO_2_ balanced with N_2_), using the ideal adsorbed solution theory (IAST), shows that at 1 bar and 303 K, the CO_2_/N_2_ selectivity is 11.5. In summary, this work reports essential data for the design of adsorption-based processes for CO_2_ capture using a Co_3_(ndc)_3_(dabco) MOF, such as pressure swing adsorption (PSA).

## 1. Introduction

The urgency to control climate change is increasing every day. At this point, a combination of approaches and technologies is needed to reduce atmospheric greenhouse gas concentrations. Further developments in sustainable and green energy sources, improvement in energy efficiency, and advancements in carbon capture for storage or utilization (CCS/CCU) are needed [1]. Adsorption processes are a promising approach for several of these necessary developments. Adsorption can be used for carbon dioxide (CO_2_) capture from flue gases and biogas upgrading [2,3,4,5,6]. Several porous materials have been evaluated for such applications, including activated carbons [7,8,9], zeolites [10,11], amine-modified solid supports [12,13], and more. Furthermore, in the last decade, metal–organic frameworks (MOFs) have been extensively studied as adsorbents in CO_2_ removal applications [14,15,16,17,18].

MOFs are porous crystalline materials in which the porous network is based on the linkage of metal centers and organic moieties [19]. This rationale behind MOF structures allows for the design and synthesis of an almost unlimited number of morphologies. Additionally, pore size tuning and functionalization are straightforward [20]. This positions MOF materials as a unique class of porous solids suitable not only for gas separation but also for other applications such as catalysis, drug delivery, and sensors [21,22,23,24,25,26].

**Figure 1:**
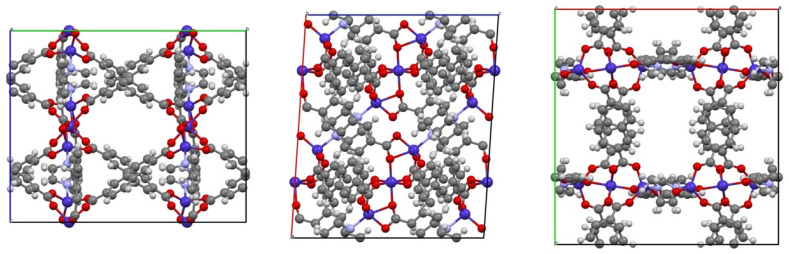
View of the unit cell of Co_3_(ndc)_3_(dabco) along the three crystallographic axes, a, b, and c, respectively (Co: blue, O: red, C: grey, H: white, and N: light blue). Reprinted from [28], with permission from Elsevier.

**Figure 2:**
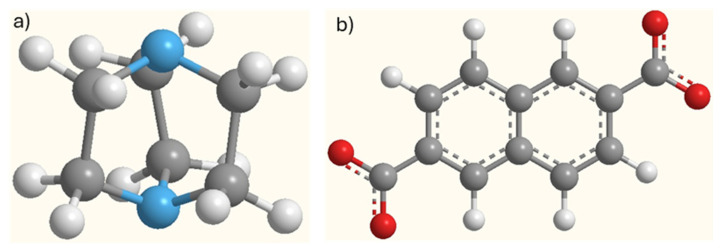
Representation of ligands (**a**) dabco: 1,4-diazabicyclo[2.2.2]octane and (**b**) ndc: 2,6-naphthalenedicarboxylate (O: red, C: grey, H: white, and N: light blue).

In this work, we study the adsorption equilibrium of CO_2_ and nitrogen (N_2_) on the scarcely studied Co_3_(ndc)_3_(dabco) MOF. This porous material has high surface area, narrow channels, and high thermal stability [27]. Previous reports have shown that it has high adsorption capacity for hydrogen (H_2_) and methane (CH_4_) storage [17,27]. Co_3_(ndc)_3_(dabco) consists of a variation of the primitive cubic net with three-dimensionally connected pores (ndc: 2,6-naphthalenedicarboxylate; dabco: 1,4-diazabicyclo[2.2.2]octane), see Figure 1 and Figure 2.

Here, we present the adsorption equilibrium isotherms of CO_2_ and N_2_ at 273 K, 303 K, and 323 K on Co_3_(ndc)_3_(dabco) up to 35 bar. Furthermore, we fit the Langmuir isotherm model to the experimental adsorption data and estimate the CO_2_/N_2_ selectivity for typical flue gas streams using the ideal adsorbed solution theory (IAST). The isosteric heat of adsorption is calculated from the experimental adsorption equilibrium data.

## 2. Results and Discussion

**Figure 3:**
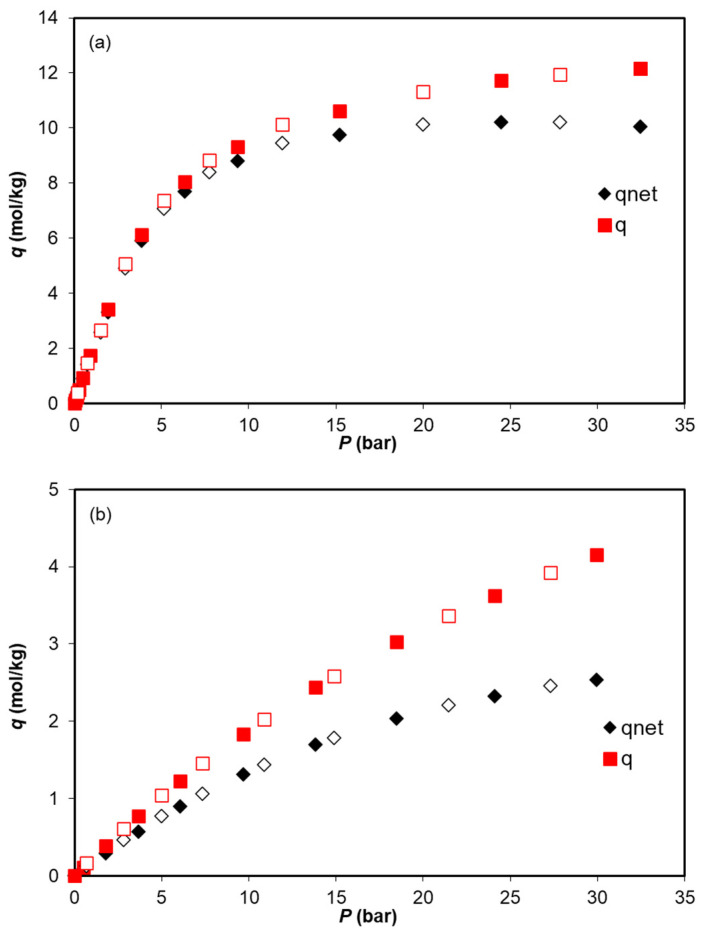
Adsorption equilibrium isotherms of (**a**) CO_2_ and (**b**) N_2_ on the Co_3_(ndc)_3_(dabco) MOF at 303 K, reported in terms of net ($q_{\mathrm{net}}$) and absolute (q) amounts adsorbed. The filled and empty symbols denote the adsorption and desorption data, respectively.

The single-component adsorption equilibrium of CO_2_ and N_2_ on Co_3_(ndc)_3_(dabco) was measured at 273 K, 303 K, and 323 K and up to 35 bar. The adsorption equilibrium data are first interpreted in terms of the net amount adsorbed, $q_{\mathrm{net}}$, and subsequently converted to the absolute amount adsorbed, q, using the specific volumes of the porous space and solid matrix ($v_{p}=0.77$ cm^3^/g; $v_{s}=0.58$ cm^3^/g), which were obtained from N_2_ physisorption at 77 K and He picnometry at 323 K, respectively [28]. The net and absolute amounts adsorbed for CO_2_ and N_2_ are listed in Table 1 and Table 2. The visualization of both adsorption quantities is exemplified in Figure 3 for CO_2_ and N_2_ adsorption at 303 K. The results show that in the low-pressure region, $q_{\mathrm{net}}$ and q are equivalent but diverge in the high-pressure region. This illustrates the well-known importance of conversion to absolute amounts, especially from medium to high pressures.

**Figure 4:**
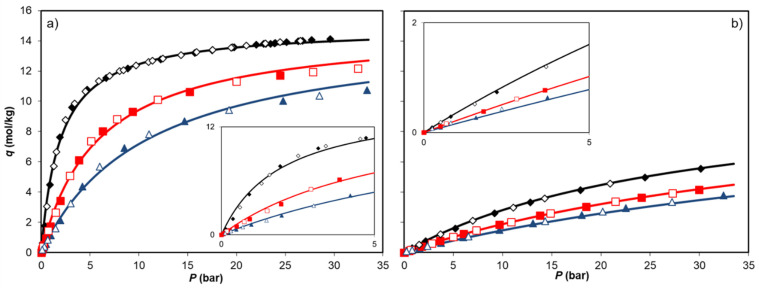
Adsorption equilibrium isotherms of CO_2_ (**a**) and N_2_ (**b**) on Co_3_(ndc)_3_(dabco) MOF at 273 K (♦), 303 K (■), and 323 K (▲). The filled and empty symbols denote the adsorption and desorption data, respectively. The solid lines represent the Langmuir model fitting.

The CO_2_ and N_2_ adsorption equilibrium isotherms, reported in absolute amount adsorbed, are shown in Figure 4a and Figure 4b, respectively, and display the measured adsorption and desorption data. The experimental data indicate the absence of hysteresis and show that both gases exhibit classic Langmuirian Type I isotherms according to the physisorption isotherm classification recommended by IUPAC [29]. This reflects the behavior of a microporous adsorbent material, as is the case of the MOF under study and was reported in our previous study [28]. N_2_ adsorption (77 K) on this MOF sample demonstrated its porosity falls entirely in the micropore range, presenting a specific surface area of 1460 m^2^/g and pore volume of 0.77 cm^3^/g [28].

The CO_2_ adsorption isotherms are steeper in the Henry law region than the N_2_ isotherms, and the CO_2_ adsorption capacity is much greater in the high-pressure region. Thus, the MOF demonstrates high CO_2_ adsorption capacities at low pressures, an important feature for post-combustion carbon capture applications. The N_2_ adsorption isotherms are much closer to linearity and present lower adsorption capacities than those of CO_2_.

The Langmuir adsorption model was successfully fitted to the experimental adsorption equilibrium data, as shown in Figure 3. This figure demonstrates that the Langmuir model, which can be taken as one of the simplest nonlinear adsorption isotherm models, effectively describes the experimental data at both low and high pressures and can be employed in process modeling and simulation. The parameters obtained from the Langmuirian fitting and the average relative error (ARE) are presented in Table 3.

**Figure 5:**
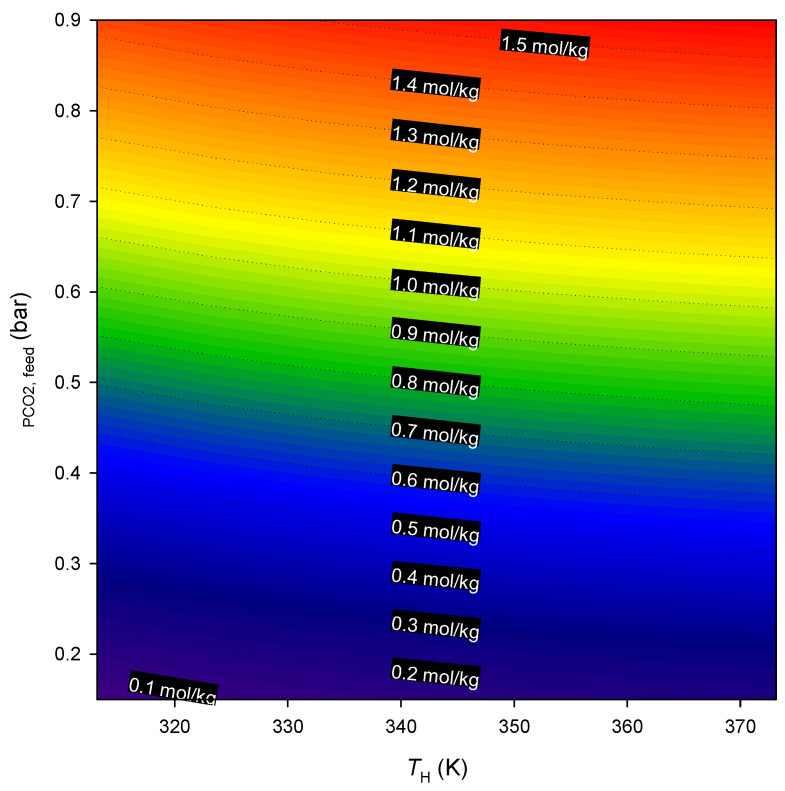
Contour plots of the CO_2_ working capacity as a function of desorption temperature, *T*_H_, and CO_2_ partial pressure in the feed stream (*P*_CO_2_,feed_). The adsorption temperature is 303 K (c.a. ambient temperature) and the desorption pressure is 0.15 bar (CO_2_ partial pressure under atmospheric pressure).

A preliminary evaluation of the potential of Co_3_(ndc)_3_(dabco) for CO_2_ capture can be based on the theoretical net amount adsorbed under different feed and regeneration conditions (pressure/temperature) that emulate adsorption processes based on pressure and temperature swings. Figure 5 shows contour plots of the CO_2_ working capacity as a function of the CO_2_ feed pressure and selected desorption temperature. The working capacity is defined as the CO_2_ amount adsorbed in the feed conditions minus the amount that remains adsorbed at desorption conditions. It should be noted that the amounts adsorbed at higher temperatures were obtained by extrapolating the adsorption equilibrium measured at 273–323 K using the previously fitted Langmuir isotherm model. The results indicate that the CO_2_ pressure at feed conditions significantly impacts the working capacity; on the other hand, the temperature appears to have a more moderate effect on the CO_2_ working capacity. For example, using a high (desorption) temperature of 373 K and a CO_2_ feed pressure of 0.45 bar, the CO_2_ working capacity is ∆q=0.76 mol/kg. This value can be significantly increased (to ∆q=1.58 mol/kg) by doubling *P*_CO2,feed_ to 0.9 bar (equivalent to a total pressure of 6 bar for a flue gas with 15%-mol CO_2_). Additionally, if the desorption temperature is the same as the feed temperature (303 K), the CO_2_ working capacity, swinging between 0.9 and 0.15 bar of CO_2_, would be 1.38 mol/kg. This demonstrates that pressure swings provide a much more effective approach than temperature swings for regenerating Co_3_(ndc)_3_(dabco) when adsorbing CO_2_.

**Figure 6:**
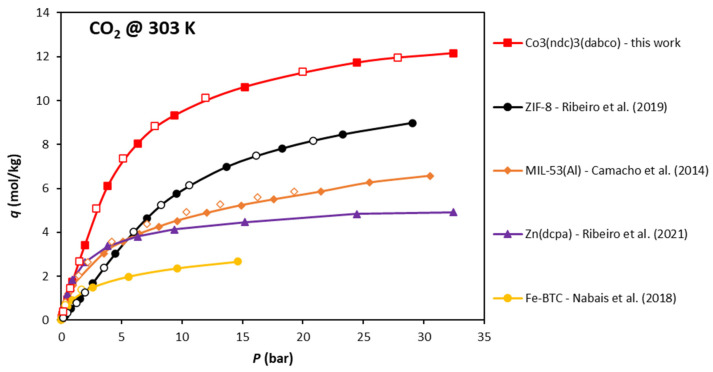
CO_2_ adsorption equilibrium isotherm at 303 K on CO_3_(ndc)_3_(dabco), ZIF-8 [23], MIL-53 [14], Zn(dcpa) [18], and Fe-BTC [30].

Figure 6 shows a comparison between the adsorption equilibrium isotherm of CO_2_ on Co_3_(ndc)_3_(dabco) at 303 K with other MOF materials evaluated by our group under the same thermodynamic conditions. The MOF under study in this work presents a significantly higher adsorption capacity at the high-pressure limit, followed by ZIF-8 [ZIF: Zeolitic imidazolate framework], MIL-53(Al) [MIL: Materials Institute Lavoisier], Zn(dcpa) [dcpa: 2,6-dichlorophenylacetate], and Fe-BTC [BTC: benzene-1,3,5-tricarboxylate]. This behavior is due to the large specific pore volume of CO_3_(ndc)_3_(dabco), 0.77 cm^3^/g. This highlights the great potential of this MOF not only for CO_2_ separation from gas mixtures but also for adsorptive storage of large amounts of CO_2_.

**Figure 7:**
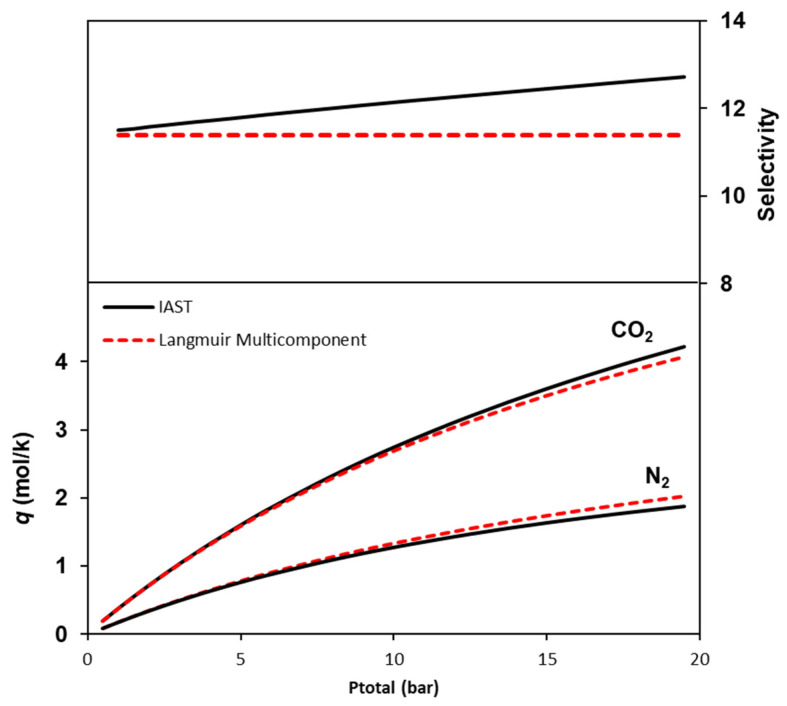
Competitive adsorption equilibrium isotherms for a 15%/85% CO_2_/N_2_ mixture and equilibrium selectivity at 303 K. solid lines: IAST model; dashed lines: multicomponent Langmuir model.

The ideal adsorbed solution theory (IAST) is employed to predict the adsorption behavior of CO_2_/N_2_ mixtures. The potential of Co_3_(ndc)_3_(dabco) for use in CO_2_/N_2_ separations can be initially evaluated by comparing the multicomponent adsorption equilibria predicted from the single-component measurements. Figure 7 presents the binary adsorption equilibrium results predicted for 303 K and 1 bar using IAST and the straightforward extension of the Langmuir model to multicomponent adsorption. Both the IAST and extended Langmuir isotherm model predict similar competitive adsorption equilibrium isotherms. However, slight differences in the equilibrium data result in different selectivity values: 11.4 for the Langmuir model and between 11.4 e 12.7 for the IAST predictions.

The isosteric heat of adsorption, $Q_{\mathrm{st}}$, was calculated from the experimental adsorption equilibrium data through the Clausius–Clapeyron equation [31]: ${\left(\log\;P\right)}_{q}=\mathrm{const}-Q_{\mathrm{st}}/RT$, where R is the ideal gas constant, T the system temperature, and P the partial pressure of the adsorptive in the gas phase. According to this relationship, a plot of log P versus −1/RT, at constant loading q, should yield a straight line with a slope giving the value of $\;Q_{\mathrm{st}}.$ Since the experimental adsorption data are never obtained at constant loading, for the purpose of calculating $Q_{\mathrm{st}}$, the adsorption equilibrium isotherms were fitted to polynomials to determine the pressure values corresponding to the same loading amounts.

**Figure 8:**
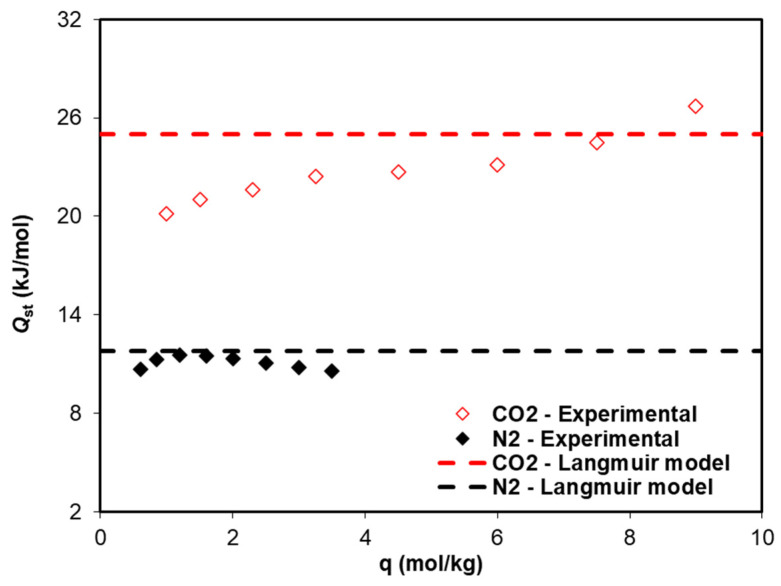
Isosteric heat of adsorption, *Q*_st_, as a function of the CO_2_ and N_2_ loadings. The symbols represent the *Q*_st_ values calculated from the isosteric plot of the experimental adsorption equilibrium data. The dashed lines represent the *Q*_st_ values obtained from the fitting with the Langmuir model.

The CO_2_ and N_2_ isosteric heats of adsorption as a function of adsorbate loading are plotted in Figure 8. The results show that for CO_2_, $Q_{\mathrm{st}}$ increases with the loading from approximately 20 to 27 kJ/mol, while for N_2_, the value remains almost constant at 11 ± 0.5 kJ/mol. The values determined from the experimental data are consistent with those obtained from the Langmuir fitting of the Langmuir adsorption isotherm model, which are 25.0 kJ/mol for CO_2_ and 11.8 kJ/mol for N_2_.

## 3. Materials and Methods

The Co_3_(ndc)_3_(dabco) sample under study was supplied by the Materials Center at the Technical University Dresden (Dresden, Germany). Its detailed characterization, including powder X-ray diffraction (PXRD), thermogravimetric analysis (TGA), N_2_ physisorption at 77 K, and helium porosimetry, is reported elsewhere [17,28]. The gases used were provided by Air Liquide (Algés, Portugal) with purity of 99.998% (CO_2_) and 99.995% (N_2_).

Single-component adsorption equilibrium isotherms of CO_2_ and N_2_ up to 35 bar were measured at 273 K, 303 K, and 323 K using a high-pressure magnetic-suspension microbalance ISOSORP 2000 (Rubotherm GmbH, Bochum, Germany) [14,32]. The Rubotherm balance has a resolution of 10^−5^ g, uncertainty ≤ 0.002%, and reproducibility ≤ 3 × 10^−5^ g for a maximum load of 25 g. The temperature inside the measurement chamber (containing the MOF sample) is controlled (±0.1 K) with a Julabo GmbH thermostatic bath F32 HL (Seelbach, Germany). The pressure measurements are made using three pressure transducers with different ranges to ensure good measurement accuracy at all evaluated pressures. For pressure up to 1 bar, a Baratron model 627D (MKS Instruments GmbH, Munich, Germany), with accuracy of 0.12% of the measured value, is used. Within the 0–10 bar and 10–35 bar ranges, Omegadyne Inc. (Sunbury, OH, USA) models PX01C1-150A5T and PX01C1-500A5T are used, respectively (both with an accuracy of 0.05% of the full scale). The data are monitored and recorded using an in-house developed software.

The adsorption equilibrium data were determined using a MOF sample of about 600 mg, with several steps of gas supply to the measurement chamber. Upon reaching the maximum pressure of the isotherm being measured, sequential depressurization steps were also performed to collect both adsorption and desorption data, allowing for the evaluation of hysteretic effects. Detailed information about the apparatus and procedure employed here are reported elsewhere [32].

The Co_3_(ndc)_3_(dabco) sample was received already activated and stored under an argon atmosphere. Therefore, prior to the adsorption measurements, the pre-treatment of the sample was limited to vacuum overnight at the highest temperature of measurement (323 K). The synthesis and characterization of the Co_3_(ndc)_3_(dabco) was previously detailed by Ribeiro et al. [17,28].

Adsorption equilibrium data determined experimentally can be reported in terms of different quantities, namely, net, excess, and absolute amounts [33,34]. Here, the interpretation of the experimental data is first carried out in terms of net adsorption, as this approach allows for presenting the amount adsorbed without relying on a reference state, which is typically established using (nearly) nonadsorbing probe molecules (e.g., helium). The net amount adsorbed per mass of adsorbent, $q_{\mathrm{net}}$, is determined by

(1) $$q_{\mathrm{net}}=\frac{w-m_{s}-m_{h}+V_{h}\rho_{g}}{m_{s}}\;,$$

where w is the apparent mass measured in the balance, $m_{s}$ is the mass of adsorbent employed, $m_{h}$ and $V_{h}$ are the mass and volume of all physical parts in the measuring cell that contribute to buoyancy effects, and $\rho_{g}$ is the gas density at the equilibrium pressure and temperature.

Upon determining $q_{\mathrm{net}}$, the absolute amount adsorbed, *q*, can be obtained from

(2) $$q=q_{\mathrm{net}}+\left(v_{p}+v_{s}\right)\rho_{g},$$

where $v_{p}$ is the specific pore volume of the adsorbent, and $v_{s}$ is the specific volume of its solid matrix, i.e., the volume impenetrable to the adsorbate molecules ($v_{s}=1/\rho_{s}$, where $\rho_{s}$ is the skeletal density of the adsorbent). The skeletal density was previously determined through helium picnometry, corresponding to the density of the effective solid phase in which the adsorbate molecules cannot penetrate [28].

After measuring the adsorption equilibrium isotherms and converting the data to absolute adsorption, the data were fitted using the Langmuir model, which can be described as follows:

(3) $$q=\frac{q_{s}bP}{1+bP}.$$

Here, q is the amount adsorbed, $q_{s}$ is the maximum (saturation) loading, P is the pressure, and b is the adsorption affinity constant, whose temperature dependence is described as

(4) $$b=b_{0}exp\left(\frac{-Q}{RT}\right),$$

where $b_{0}$ is the pre-exponential factor, Q is the average heat of adsorption, R is the ideal gas constant, and T is the temperature.

The Langmuir model is fitted by minimizing the average relative error (ARE) between the fitted and experimental values, defined as

(5) $$ARE(\%)=\frac{100}{N_{exp}}\sum \frac{\left|q_{fit}-q_{exp}\right|}{q_{exp}},$$

where $q_{fit}$ and $q_{exp}$ are the fitted and experimental values, respectively, and $N_{exp}$ is the number of experimental points of the isotherms.

After fitting the Langmuir model to the experimental adsorption isotherms, the ideal adsorbed solution theory (IAST) was applied to extract selectivity predictions for the CO_2_/N_2_ separation. The calculations were performed using Matlab. IAST approximates a real adsorbed phase of *N* components as an ideal mixture of pure adsorbed gases at constant surface potential [35,36]. The spreading pressure, π, of the gas mixture is considered a dependent variable, just like temperature or partial pressure of each adsorbate; π is defined as

(6) $$\frac{\pi A}{RT}=\int_{0}^{p_{i}^{0}}q_{i}^{0}\left(p,T\right)dp\;\;\;\;\;\;\;\;\;\;\;\left(const\;T\right),$$

where i is the component index, A is the surface area of the adsorbent, R is the ideal gas constant, and $q_{i}^{0}$ is the adsorption isotherm of pure component *i* at the system temperature T. Under equilibrium conditions, $p_{i}^{0}$ is the hypothetical pressure at which the adsorption of pure component i yields the same value of π as that of the mixture at temperature *T*.

For an ideal adsorbed solution, the relationship between bulk and adsorbed phases can be expressed by the analog of Raoult’s law:

(7) $$p_{i}=y_{i}P=x_{i}p_{i}^{0},$$

(8) $$\sum_{j}x_{j}=1,$$

where $y_{i}$ is the mole fraction of component *i* in the gas phase, and $x_{i}$ is its mole fraction in the adsorbed phase, derived from the values of π, temperature, and partial pressures. The total amount adsorbed, q, is calculated as follows:

(9) $$\frac{1}{q}=\sum_{i}\frac{x_{i}}{q_{i}^{0}(p_{i}^{0},T)}\;,$$

from which the amount of component *i* adsorbed is easily determined:

(10) $$q_{i}=x_{i}q.$$

## 4. Conclusions

The adsorption equilibrium of CO_2_ and N_2_ on Co_3_(ndc)_3_(dabco) MOF at 273–323 K and up to 35 bar was experimentally determined. The isotherms were successfully described by the Langmuir model, and the heats of adsorption determined through the Clausius–Clapeyron equation are in the range 20–27 kJ/mol for CO_2_ and 10.5–11.5 kJ/mol for N_2_. Co_3_(ndc)_3_(dabco) has a CO_2_ working capacity of 1.58 mol/kg for adsorption and desorption conditions of 0.9 bar/303 K and 0.15 bar/373 K, respectively. The selectivity predicted by IAST for a CO_2_/N_2_ mixture of 15%/85% at 1 bar and 303 K is 11.5.

The data presented and analyzed in this work demonstrate the suitability of Co_3_(ndc)_3_(dabco) as a CO_2_ adsorbent that can be employed in pressure swing adsorption processes.

## Figures and Tables

**Table 1:** Experimental CO_2_ adsorption equilibrium data at 273 K, 303 K, and 323 K. The reference state corrections for absolute amount adsorbed (q) are $v_{p}=0.77\;{\mathrm{cm}}^{3}$/g and $v_{s}=0.58\;{\mathrm{cm}}^{3}/g$, where $v_{p}$ is the specific pore volume of the adsorbent, and $v_{s}$ is the specific volume of its solid matrix.

T = 273 K			T = 303 K			T = 323 K		
P (bar)	$q_{\mathrm{net}}$ (mol/kg)	q (mol/kg)	P (bar)	$q_{\mathrm{net}}$ (mol/kg)	q (mol/kg)	P (bar)	$q_{\mathrm{net}}$ (mol/kg)	q (mol/kg)
0.04	0.13	0.13	0.06	0.11	0.11	0.07	0.08	0.09
0.15	0.61	0.62	0.13	0.23	0.24	0.23	0.26	0.27
0.38	1.74	1.76	0.27	0.49	0.50	0.49	0.55	0.58
0.91	4.43	4.48	0.50	0.91	0.93	0.98	1.09	1.14
1.92	7.51	7.63	0.93	1.69	1.74	1.97	2.04	2.14
3.16	9.41	9.60	1.95	3.31	3.42	4.21	4.15	4.36
4.73	10.47	10.76	3.86	5.91	6.12	8.52	6.47	6.91
6.66	11.14	11.55	6.31	7.69	8.04	14.66	7.90	8.68
8.87	11.61	12.18	9.36	8.79	9.32	24.79	8.65	10.04
12.26	11.98	12.78	15.20	9.74	10.62	33.37	8.79	10.74
15.73	12.14	13.20	24.46	10.23	11.73	28.48	8.76	10.37
17.50	12.19	13.38	32.46	10.06	12.16	19.22	8.38	9.43
19.75	12.19	13.57	27.81	10.21	11.95	11.04	7.24	7.82
22.03	12.17	13.75	19.97	10.13	11.32	6.02	5.37	5.68
22.80	12.15	13.79	11.94	9.44	10.12	3.02	3.10	3.25
22.99	12.14	13.80	7.75	8.41	8.84	1.48	1.53	1.60
23.49	12.10	13.80	5.13	7.08	7.36	0.70	0.83	0.86
23.89	12.08	13.83	2.93	4.91	5.07	0.31	0.39	0.41
23.99	12.11	13.86	1.49	2.58	2.66	0.12	0.18	0.19
25.11	12.06	13.92	0.74	1.42	1.46			
26.34	12.00	13.98	0.18	0.38	0.39			
26.78	11.99	14.01						
27.71	11.92	14.05						
29.62	11.79	14.12						
26.54	12.00	14.00						
24.46	12.11	13.90						
22.97	12.16	13.82						
21.02	12.20	13.69						
19.57	12.21	13.57						
19.48	12.19	13.55						
17.56	12.21	13.41						
15.93	12.19	13.26						
14.34	12.11	13.06						
12.46	12.02	12.83						
11.39	11.94	12.68						
11.01	11.90	12.61						
9.73	11.76	12.38						
8.41	11.54	12.06						
8.04	11.49	12.00						
7.03	11.26	11.70						
5.92	10.97	11.34						
4.54	10.38	10.66						
3.41	9.64	9.85						
2.51	8.63	8.78						
1.58	6.56	6.66						
1.31	5.63	5.71						
0.61	3.01	3.04						

**Table 2:** Experimental N_2_ adsorption equilibrium data at 273 K, 303 K, and 323 K. The reference state corrections for absolute amount adsorbed (q) are $v_{p}=0.77\;{\mathrm{cm}}^{3}$/g and $v_{s}=0.58\;{\mathrm{cm}}^{3}/g$, where $v_{p}$ is the specific pore volume of the adsorbent, and $v_{s}$ is the specific volume of its solid matrix.

T = 273 K			T = 303 K			T = 323 K		
P (bar)	$q_{\mathrm{net}}$ (mol/kg)	q (mol/kg)	P (bar)	$q_{\mathrm{net}}$ (mol/kg)	q (mol/kg)	P (bar)	$q_{\mathrm{net}}$ (mol/kg)	q (mol/kg)
0.25	0.08	0.09	0.01	0.003	0.003	0.01	0.004	0.004
0.81	0.24	0.29	0.50	0.09	0.110	0.51	0.070	0.090
2.21	0.60	0.74	1.81	0.29	0.390	1.58	0.190	0.270
5.21	1.26	1.57	3.66	0.57	0.770	3.74	0.450	0.640
9.18	2.03	2.58	6.05	0.90	1.220	6.01	0.690	0.990
12.81	2.54	3.30	9.68	1.31	1.830	9.67	1.020	1.500
17.93	3.06	4.14	13.80	1.70	2.440	13.09	1.280	1.940
24.45	3.49	4.96	18.49	2.03	3.030	18.03	1.580	2.480
30.13	3.74	5.55	24.10	2.33	3.620	22.52	1.800	2.930
20.95	3.29	4.55	29.96	2.54	4.150	32.46	2.140	3.770
14.28	2.71	3.57	27.28	2.46	3.920	27.22	1.980	3.340
11.08	2.31	2.98	21.46	2.21	3.360	20.18	1.690	2.700
6.99	1.67	2.09	14.87	1.78	2.580	14.32	1.350	2.070
3.70	0.98	1.20	10.89	1.44	2.020	6.54	0.740	1.070
1.54	0.42	0.52	7.32	1.07	1.460	2.36	0.310	0.430
0.51	0.16	0.19	5.00	0.77	1.040	0.81	0.140	0.180
			2.80	0.46	0.610	0.26	0.060	0.070
			0.68	0.13	0.160			

**Table 3:** Langmuir adsorption isotherm model fitting parameters for CO_2_ and N_2_ on Co_3_(ndc)_3_(dabco).

	CO_2_	N_2_
$q_{s}$ (mol/kg)	14.9	11.0
$b_{0}$ (bar^−1^)	8.47 × 10^−6^	1.09 × 10^−4^
*Q* (kJ/mol)	25.00	11.78
ARE (%)	7.3	5.6

## Data Availability

The data are contained within the article.
